# NMR characterisation of biopolymers and lipids from hemp pomace treated with *Thermomyces lanuginosus*

**DOI:** 10.1038/s41598-026-41682-1

**Published:** 2026-03-13

**Authors:** Jelena Parlov Vuković, Tomislav Jednačak, Predrag Novak, Dora Bjedov, Marina Tišma

**Affiliations:** 1 NMR Centre, Ruđer Bošković Institute, Bijenička Cesta 54, 10000 Zagreb, Croatia; 2 Department of Chemistry, Faculty of Science, University of Zagreb, Horvatovac 102a, 10000 Zagreb, Croatia; 3 Department of Biology, Josip Juraj Strossmayer University of Osijek, Ul. Cara Hadrijana 8/A, 31000 Osijek, Croatia; 4 Faculty of Food Technology Osijek, Josip Juraj Strossmayer University of Osijek, F. Kuhača 18, 31000 Osijek, Croatia

**Keywords:** Hemp-derived products, Hemp pomace, Solid-state fermentation, *Thermomyces lanuginosus*, NMR spectroscopy, Biotechnology, Environmental sciences, Chemistry

## Abstract

The influence of *Thermomyces lanuginosus* on the chemical structure of biopolymers and lipids from hemp pomace during solid-state fermentation (SSF) was studied using Nuclear Magnetic Resonance (NMR) spectroscopy. The samples of hemp pomace before, during and after SSF were used for the isolation of biopolymers (lignin, cellulose, hemicellulose) and lipids, which were subsequently analyzed using solution and solid-state NMR spectroscopy. It was observed that SSF significantly alters the composition and quality of individual biopolymers. An increase in the carbonyl group content in lignin was noted. In lipid samples, a significant reduction in mono-, di-, and triglycerides occurred accompanied by an increase in glycerol and unsaturated fatty acids. SSF also notably impacted phosphorus-containing compounds in the hemp pomace. These results demonstrate that SSF enables targeted modification of key chemical components in hemp pomace. By altering the structure of lignin, breaking down complex lipids, and affecting phosphorus-containing compounds, SSF improves the chemical quality and functionality of the biomass. This highlights its potential as an effective method for upgrading agricultural residues into more valuable and versatile materials supporting sustainable biorefinery applications.

**Supplementary Information:**

The online version contains supplementary material available at 10.1038/s41598-026-41682-1.

## Introduction

Lignocellulosic biomass plays a pivotal role in the global shift away from fossil resources, as it could provide a sustainable alternative resource for the production of various bio-based products^1,2^. It mainly originates from agriculture, forestry, and the food industry, with data indicating that approximately 182 million tonnes of various lignocellulosic materials are available worldwide each year^2,3^. Lignocellulosic biomass can be edible, such as crops, which are commonly referred to as primary biomass, but it can also consist of industrial residues and by-products, known as secondary biomass. These materials exhibit complex chemical interactions among their constituent polymers, such as lignin, cellulose and hemicellulose, which contribute to their structural integrity and recalcitrance^4^. Recalcitrance, structural complexity, heterogeneity, voluminosity and seasonal character influence their suitability for industrial applications while their storage, transport, pretreatment and conversion technologies into valuable bio-based products are challenging. To overcome the challenges related to the pretreatment and conversion processes, a variety of methods have been developed, including chemical, physical, biological, and hybrid approaches, all designed to efficiently disrupt the complex interactions between main biopolymers, lignin, cellulose, and hemicellulose^5^.

The global industrial hemp industry is experiencing rapid growth which has led to an increased availability of hemp-derived products, by-products and process residuals^6^. Hemp pomace is a by-product of hemp oil production and can be therefore classified as secondary biomass. Derived from the fibrous remains after hemp seed and oil extraction, besides lignocellulosic polymers which include insoluble fibre (28–41%), hemp pomace contain significant amount of lipids (25–35%) and proteins (20–25%), and lower amounts of sugars (1–4%)^7,8^. Hemp fibres are composed of cellulose (39–61%), hemicellulose (11–15%) and lignin (8–16%)^8^. While most of lignocellulosic biomass usually contains low content of lipids, oil pomaces are an exception, often exhibiting high lipid content. This quantity is largely influenced by the extraction technology used during oil production^9–11^. Lipids are the key components of the plant cell membrane that form the bilayer structure, protect it and maintain integrity, contributing to its physical properties, such as flexibility, resistance to mechanical stress, and water retention. They serve as a reserve energy source. Additionally, they are found in the cuticular layer of plant cells, which acts as a protective barrier against water loss and pathogen invasion. These lipids, primarily composed of long-chain fatty acids and alcohols, enhance the hydrophobicity of the plant surface and influence the plant’s interactions with the environment. The fatty acids in lipids can also be converted into biofuels or utilized in other industrial applications, such as production of biodegradable lubricants, surfactants, cosmetics, and bioplastics. The composition of lipids can influence the yield and quality of obtained products^12^.

In this study, the effect of biological treatment of hemp pomace with *Thermomyces lanuginosus* via SSF on the chemical composition of individual biopolymers (lignin, cellulose, and hemicellulose) and lipids was investigated. Biopolymers and lipids were isolated from the fermented material at various time points and subsequently analyzed using NMR. *Thermomyces lanuginosus* was selected for this study due to its well-documented ability to produce xylanase and lipase. Its strong lipolytic activity makes it especially suitable for modifying the lipid content of hemp pomace, as demonstrated in our previous studies^13,14^.

The aim was to gain a deeper understanding of how fungal activity influences the structural integrity and properties of biopolymers, as well as the extent of lipid hydrolysis, leading to the formation of potentially value-added compounds such as fatty acids and glycerol.

NMR spectroscopy has been widely employed for over three decades as a powerful tool for the structural and compositional analysis of lignocellulosic biomass, both in solution and in the solid-state. Its ability to provide detailed qualitative and quantitative information on functional groups, molecular architecture, and the degree of polymerization makes it particularly well-suited for investigating complex biopolymer systems^15–17^. In the context of this study, NMR was chosen due to its capacity to non-destructively elucidate subtle structural changes in lignin, cellulose, and hemicellulose, as well as to monitor the transformation of lipid components during fungal fermentation.

This is the first report to use solution and solid-state NMR spectroscopy to monitor the time-dependent chemical changes in lignin, cellulose, hemicellulose, and lipid fractions during fungal treatment of hemp pomace. The findings provide novel insights into the structural modifications induced by fungal activity, highlighting the potential to expand the industrial valorization of hemp pomace beyond its traditional use as animal feed.

## Materials and methods

### Substrate and microorganism

Hemp pomace (HP), a by-product of cold-pressed hemp oil manufacturing was obtained from the Konopko Ltd., Slovenia. Prior to SSF, the HP underwent milling with an ultracentrifugal mill (ZM200, Retsch, Germany) to obtain particles smaller than 5 mm.

Thermophilic fungus *Thermomyces lanuginosus* Tsiklinsky (ATCC76323) was sourced from the DSMZ-German Collection. The fungus was incubated for 10 days at 45 °C using potato dextrose agar (PDA) in Petri dishes prior being used in SSF.

### Chemicals

Sulphuric acid was obtained from T.T.T. d.o.o (Sveta Nedelja, Croatia), sodium hydroxide from Kefo (Sisak, Croatia), ethylenediaminetetraacetic acid (EDTA) from Sigma-Aldrich (Missouri, USA), methanol from J.T.Baker (New Jersey, USA), chloroform from Carlo-Erba (Val de Reuil, France), ethanol and acetone from Gram-mol (Zagreb, Croatia), acetic acid from Lach-Ner (Zagreb, Croatia), PDA from Biolife (Milan, Italy), CDCl_3_ and D_2_O from Euriso-Top (Saclay, France).

### Solid-state fermentation

SSF was conducted at 45 °C in 0.65 L laboratory jars in an incubator with the air fan set on 20% (KB 115, BINDER GmbH, Germany). 50 g of HP was weighed, mixed with 70 mL of distilled water, and sterilized at 121 °C for 15 min. The inoculum was prepared by suspending seven 10 mm mycelial discs in 10 mL of sterile distilled water, which was then added into each jar, covered with sterilized paper. SSF was carried out 4, 7 and 10 days. For each experimental day, triplicate samples were prepared. In total there were 9 jars. At the end of each designated day, laboratory jars were removed from the incubator, sterilized, and dried at room temperature approximately one day to a moisture content below 10%. The fermented HP substrates were used for isolation of lipids and biopolymers (lignin, cellulose and hemicellulose) and preparation of phosphorus-rich extracts.

### Isolation of lipids, biopolymers and preparation of phosphorous-rich extracts from hemp pomace prior, during and after solid-state fermentation

#### Lipid isolation

Lipids were isolated from the samples prior to and during SSF (after 4, 7 and 10 days of fermentation). A total of 4 samples of hemp oil pomace lipid extracts (HOPLE) were analysed using proton NMR spectroscopy, labelled as: 1) HopLE—prior SSF, 2) HopLE 4—after 4 days of SSF, 3) HopLE 7—after 7 days of SSF, and 4) HopLE 10—after 10 days of SSF.

Lipids were isolated according to the protocol described in already published articles^18,19^. In summary, 1.5 ± 0.1 g of the sample was weighed into a tube and combined with a solvent mixture of water, methanol, and chloroform (0.8:1:2; w/v) to the final volume of 5.7 mL (1.2 mL water, 1.5 mL methanol, and 3.0 mL chloroform). The mixture underwent ultrasonic treatment in a water bath at room temperature (23 °C) for 30 min. Following extraction, the samples were centrifuged at 1000 *g* for 20 min at 4 °C. The lipid-containing organic phase was then collected, filtered through Whatman No. 1 paper, and transferred into pre-weighed tubes. Finally, chloroform was completely evaporated by air-drying. The obtained lipid extracts were used for NMR analysis.

#### Preparation of phosphorus-rich extracts

Extract containing phosphorus was prepared prior to and during SSF (after 4, 7 and 10 days of fermentation). A total of 4 samples of hemp oil pomace phosphorus-rich extracts (HopPE) were analysed using ^31^P NMR spectroscopy, such as: 1) HopPE—prior SSF, 2) HopPE 4—after 4 days of SSF, 3) HopPE 7—after 7 days of SSF and 4) HopPE 10—after 10 days of SSF.

Preparation of extracts for ^31^P NMR spectroscopy followed the methods of Liu et al.^20^ in the way that phosphorus was extracted from 0.5 g of the sample with 30 mL of 0.5 M sodium hydroxide (NaOH) and 25 mM ethylenediaminetetraacetic acid (EDTA). The samples were incubated in a water bath with vortex agitation for 16 h at room temperature (23 °C). The extracts were then centrifuged for 30 min at 8000 *g* and filtered through Whatman No. 1 filter paper. The filtrate was used for the quantification of total dissolved phosphorus.

### Klason lignin isolation

Lignin was isolated from the samples prior to and during SSF (after 4, 7 and 10 days of fermentation). A total of 4 samples of lignin (HopLIG) were analysed using ^13^C CP MAS spectroscopy such as: (1) HopLIG—prior SSF, (2) HopLIG 4—after 4 days of SSF, (3) HopLIG 7—after 7 days of SSF and (4) HopLIG 10—after 10 days of SSF.

Lignin was isolated according to the previously published method^21^.

To isolate lignin, 1 g of hemp pomace was extracted in 80% ethanol for 30 min at 80 °C in a water bath, centrifuged at 1000 *g*, and the supernatant was decanted after each incubation. This procedure was repeated four times. In the final step, the precipitate was washed with 96% acetone and dried overnight in an oven at 70 °C. For Klason lignin determination, 100 mg of extracted tissue from each sample was weighed in triplicate and placed into 150 mL Erlenmeyer flasks, followed by the addition of 1.5 mL of 72% sulphuric acid. The samples were incubated for 1 h at 30 °C in a water bath with mixing every 15 min. After incubation, the 72% sulphuric acid was diluted with distilled water to a final concentration of 4%, and the samples were autoclaved at 121 °C for 1 h. The resulting mixture was filtered using pre-weighed filter papers, with the filtrates retained for soluble lignin determination. The residue was washed with hot distilled water until neutral pH was reached. The filter papers were then dried overnight at 70 °C to a constant weight and subsequently weighed. During Klason lignin isolation, a fraction of lignin remains soluble in sulphuric acid and is retained in the filtrate. The Klason lignin residue was collected and subsequently analysed using NMR spectroscopy.

### Cellulose isolation

Cellulose was isolated from hemp pomace according to Mohamad and Jai^22^.

A 1 g of the sample was mixed with 10 mL of a reaction mixture containing 17.7% NaOH and 10% EDTA. The mixture was heated to 100 ±  °C while stirring at 500 rpm for 30 min. Subsequently, the sample was transferred to a 50 mL Falcon tube, and 20–30 mL of distilled water was added. The tube was vortexed to ensure uniform mixing and dissolution of any residual chemicals. Centrifugation was performed at 1000 *g* to separate the solid phase from the liquid. The supernatant was carefully decanted, ensuring that the precipitate was not lost. The process of adding distilled water, vortexing, and centrifugation was repeated five times until the supernatant became clear. Finally, the extracted samples were dried in an oven at 100 ± 5 °C overnight and used for NMR characterization.

### Hemicellulose isolation

Hemicellulose was isolated from the samples according to the Rabetafika et al.^23^ 5 g of the sample was mixed with 100 mL of 1% NaOH(*w/v*) and heated to 80 °C at 500 rpm during 3 h to facilitate the dissolution of hemicellulose in the alkaline solution. Subsequently, the mixture was cooled to room temperature, and the pH was adjusted to 5–6 using acetic acid to induce precipitation of impurities and stabilize the hemicellulose. The solid fraction was separated from the liquid phase via centrifugation at 4000 rpm. The supernatant, containing dissolved hemicellulose, was carefully transferred to a clean vessel for further processing. The residual solid was washed with distilled water until neutral pH was achieved and then dried. To precipitate the hemicellulose, three volumes of 95% ethanol were gradually added to the filtrate under continuous stirring. The mixture was then left at 4 °C for 12 h to enhance precipitation. The precipitated hemicellulose was collected by centrifugation at 4000 rpm for 10 min and subsequently washed twice with 70% ethanol to remove residual impurities. Finally, the isolated hemicellulose was dried in an oven at 100 °C until a constant weight was obtained.

### Analytical methods

#### Solution-state NMR

^1^H NMR spectra of lipid extract samples and ^31^P NMR spectra of phosphorus-rich extracts were recorded using a Bruker Avance 600 NMR spectrometer with a 5 mm dual probe equipped with a z-gradient at room temperature. Proton NMR spectra of lipid extracts in chloroform-d (99.8%, Aldrich) were observed with relaxation delay of 5 s and 64 scans.

^31^P NMR spectra in D_2_O (99.8%, TCI) with spectral width of 399.66 Hz were measured with 2048 scans, FID resolution of 0.34 Hz and relaxation delay of 4.32 s.

The standard deviation was calculated from the three measured ^31^P NMR spectra of the HopPE sample (*σ*(ortophosphate) = 0.0008, *σ*(monoester) = 0.0441).

#### Solid-state NMR

Solid-state ^13^C CP MAS NMR spectra were recorded on a Bruker Avance Neo 400 spectrometer equipped with a broad band magic angle spinning (MAS) probe. The samples were spun at the magic angle in 4 mm rotors at a rotation frequency of 15 kHz. The spectra were externally referenced to glycine.

In the ^13^C CP MAS NMR spectra of the complex mixture sample, the intensities of a peak at 172.8 ppm amounted to 0.3347, 0.3405 and 0.3499, relative to the peak at 73.1 ppm, which was normalized to 1.0000. The standard deviation and the relative standard deviation were 0.0077 and 2.24%, respectively.

## Results and discussion

Solid-state fermentation is an inherently complex process due to the heterogeneous nature of the system which represents one of the major challenges related to process scale-up as well as standardization. Therefore there is the need for development of fast and accurate analytical methods for determination of chemical composition of both raw and fermented lignocellulosic biomass. To investigate the structural and compositional changes in hemp pomace during SSF, a comprehensive analysis was performed using solution and solid-state NMR spectroscopy. The composition and structural features of lignin, cellulose, hemicellulose, lipids, and phosphorus-containing compounds were examined at different fermentation time points. Through the application of NMR techniques, non-destructive and detailed characterization of molecular changes induced by fungal enzymatic activity was enabled. The transformations occurring within the lignocellulosic matrix and lipid fractions during the treatment with *Thermomyces lanuginosus* were thus monitored and evaluated. In the following sections, the observed changes in the individual components are presented and discussed with regard to fermentation progress and their potential implications for biomass valorization.

### Influence of solid-state fermentation on the structure of hemp pomace lipids and their hydrolysis products

Lipids were isolated from hemp pomace prior to and during SSF (after 4, 7 and 10 days of fermentation) and analysed by proton NMR spectroscopy. The samples are labelled as HopLE, HopLE 4, HopLE 7 and HopLE 10.

**Fig. 1:**
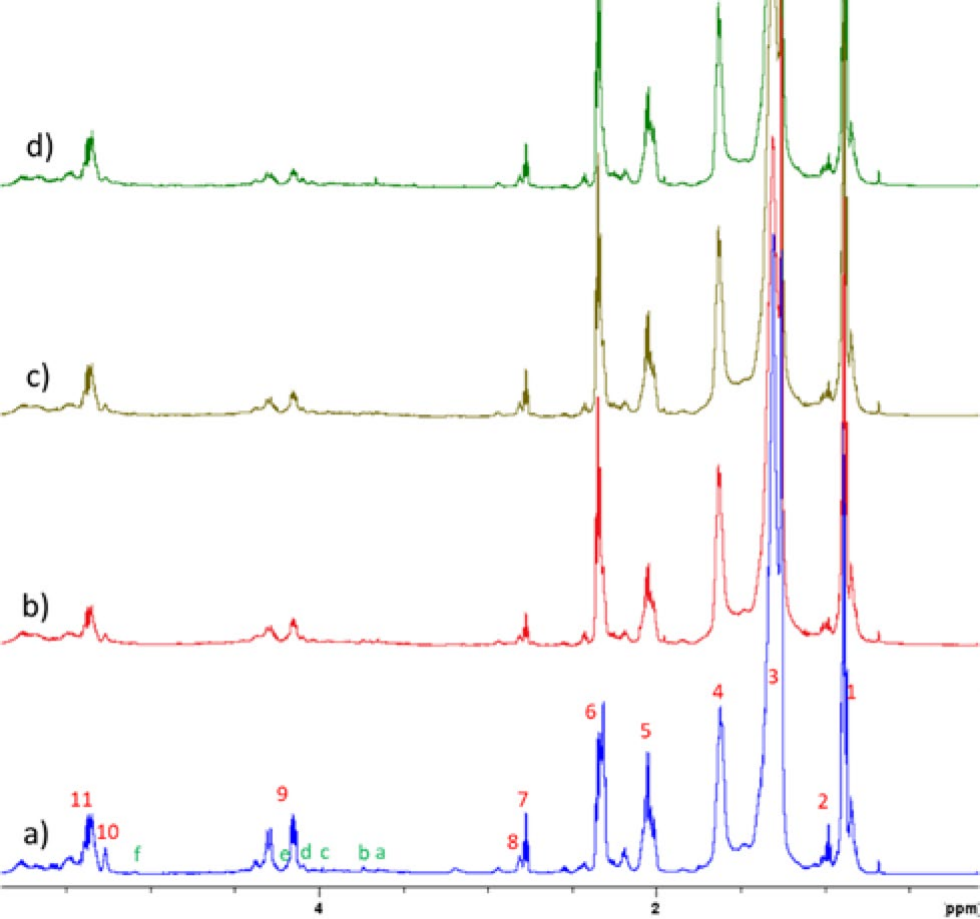
^1^H NMR spectra of hemp oil pomace lipid extracts in CDCl_3_ at 25 °C: (**a**) HopLE, (**b**) HopLE 4, (**c**) HopLE 7, and (**d**) HopLE 10.

Assignment and distribution of fatty acids and glycerides have been determined using previously established equations to calculate percentages of glycerides and fatty acids by integrating specific spectral regions. ^1^H NMR spectra of hemp oil pomace lipid extracts (HopLE, HopLE 4, HopLE 7 and HopLE 10) are shown in Fig. 1. ^1^H NMR assignments of hemp oil pomace lipid extracts^18,19^ are presented in Supplementary file (Table S1. 1H NMR assignments of hemp oil pomace lipid extracts). The results of the composition of lipids isolated from hemp pomace (triglycerides, diglycerides, fatty acids, glycerol, saturated and unsaturated fatty acids) are shown in Table 1.

**Table 1:** The composition of hemp oil pomace lipid extracts.

Composition	Hemp oil pomace lipid extract samples			
	HopLE	HopLE 4	HopLE 7	HopLE 10
N_TG_, %	68.76	43.47	44.32	55.45
N_1,2-DG_, %	15.42	14.79	11.63	3.58
N_1,3-DG_, %	10.29	24.39	9.99	2.92
N_1-MG_, %	5.53	6.53	5.93	2.26
NG, %	–	10.59	28.13	35.79
MUFA, %	43.46	56.76	61.10	60.11
PUFA (Ln, %)	9.15	8.69	7.11	5.62
PUFA (L, %)	12.74	8.21	8.20	8.53
UFA, %	65.35	73.66	76.41	74.26
SFA, %	34.65	26.34	23.59	25.76

N_TG_ ,Triglycerides; N_1,2DG_ ,1,2 diglycerides; N_1,3DG_, 1, 3- diglycerides; N_1MG_, 1- monoglycerides; NG, Glycerol; Ln, Linolenic acid; L, Linoleic acid; MUFA, Monounsaturated fatty acids; PUFA, Polyunsaturated fatty acids; UFA Unsaturated fatty acids; SFA, Saturated fatty acids.

In general, hemp seed oils are classified as polyunsaturated fatty acids rich or PUFA-rich (L + Ln) products, typically accounting 75% of the total fatty acid content whereas more than 50% of PUFA consists of linoleic acid. The methyl signals observed at 0.97 and 0.89 ppm are the most significant indicators of the presence of linolenic and other fatty acids. Integrals of both of these signals decreased upon exposure to *T. lanuginosus*. It was calculated that after 10 days of fermentation, the conversion of linolenic acid was 27.7%, while that of linoleic acid was 33.1%. The linoleic acid content in hemp pomace prior to fermentation (12.74%) is lower than the contents typically reported for hemp seed oils^24,25^. In hemp oils, linoleic acid is generally found in higher concentrations than α-linolenic acid, which was also observed in the hemp pomace used in this study, where linoleic acid (12.74%) predominated over α-linolenic acid (9.15%).

However, the omega-6 to omega-3 ratio (or n–6/n–3 PUFA ratio) was found to be nutritionally unfavourable. The ratio is a key nutritional indicator because omega-6 and omega-3 fatty acids have opposing biological roles. Omega-6 fatty acids tend to promote pro-inflammatory processes (in excess) while omega-3 fatty acids are typically anti-inflammatory and beneficial for cardiovascular, neurological, and metabolic health. Recommended ratio is usually around 4:1 to 1:1 (n–6:n–3).

The other important compounds in hemp oil are monounsaturated fatty acids (MUFA). The most common MUFA is oleic acid with portions between 12 and 19% of the total fatty acids^17,26^. The MUFA content in the HopLE sample differs significantly from those observed for oil extracted from hemp hearts, whole hemp seeds, hemp cake, hemp seed hulls and cold pressed hemp oil^27^. Surprisingly, in our study the content of MUFA in all investigated samples was 3 times higher comparing to the published results of MUFA content in hemp hearts (13.0%), whole seeds (12.0%), hemp cake (10,4%) and hemp hulls (11.1%)^27^.

Thus, the overall fatty acid composition of hemp pomace indicates its potential as a valuable raw material in sectors where an enhanced monounsaturated fatty acid (MUFA) profile is desired. This is particularly relevant for the food and beverage industry, where MUFAs are associated with improved nutritional quality and oxidative stability of products^28^, as well as in biofuel production, where MUFA-rich feedstocks contribute to favorable fuel properties such as oxidative stability, cold flow behavior, and combustion efficiency^28,29^.

Consequently, after exposing hemp pomace to *Thermomyces lanuginosus* for 10 days, a significant reduction in mono-, di-, and triglycerides was observed, accompanied by a notable increase in glycerol content, reaching up to 35%. Glycerol, a versatile and valuable compound, is primarily produced as a by-product during the biodiesel production process. However, it can also be synthesized through fermentation using various microorganisms, including fungi, as demonstrated in this study. Fungal fermentation offers an environmentally friendly and sustainable method for glycerol production, with the potential to reduce waste and enhance the economic viability of industrial processes. In addition to its use in the food, beverage, pharmaceutical, and cosmetic industries, glycerol can also be utilized in the production of bio-based polymers, antifreeze formulations, and as a humectant in personal care products^30,31^.

#### Influence of solid-state fermentation on the structure of hemp pomace phosphorus compounds

**Fig. 2:**
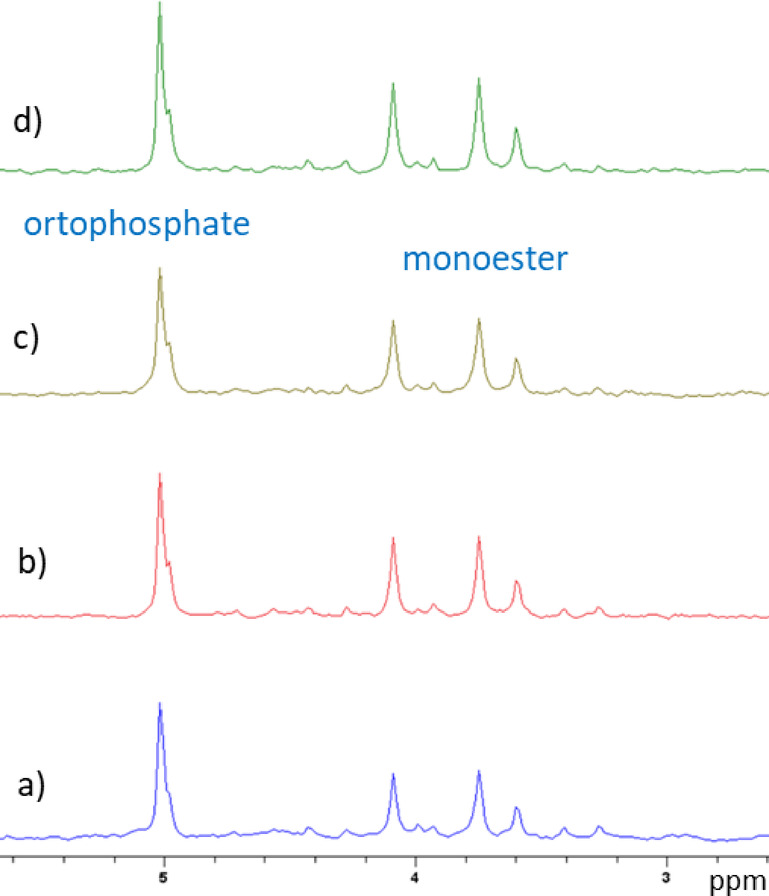
^31^P NMR spectra of hemp pomaces phosphorus-rich extracts in D_2_O at 25 °C: (**a**) HopPE, (**b**) HopPE 4*,* (**c**) HopPE 7, and (**d**) HopPE 10.

Chemical composition of the hemp pomaces after NaOH-EDTA extraction to obtain phosphorus-rich extracts has been determined by ^31^P NMR^19,20^. The characteristic spectral regions, corresponding phosphate groups and their percentages are shown in Table 2. ^31^P NMR spectra are displayed in Fig. 2.

**Table 2:** ^31^P NMR assignments and percentages of phosphorus compounds in the hemp pomace phosphorus-rich extracts.

Composition / %	Hemp oil pomace phosphorus rich extracts			
	HopPE	HopPE 4	HopPE 7	HopPE 10
Orthophosphate ( ≈ 5.20 ppm)	33.43	34.30	34.96	36.86
Monoester (3.15–5.10 ppm)	66.57	65.70	65.04	63.14

Phosphorus is a vital macronutrient for the growth and completion of the life cycle of plants. The main phosphorus components present in hemp pomaces are monoesters and ortophosphates. Monoesters belong to organic while orthophosphates belong to inorganic forms of phosphates. Although ^31^P NMR spectra look very similar (Fig. 2), the portion of monoesters and orthophosphates has changed significantly during SSF. As can be seen from the results presented in Table 2, the samples contain dominantly monoesters (δ 3.15–5.10 ppm), but during exposure to *T. lanuginosus* the orthophosphates content (≈ δ 5.20 ppm) increased, while the content of monoesters decreased. Inorganic phosphates are generally characterized by low solubility and a high sorption capacity in the soil. The results presented in Table 2 demonstrate that *Thermomyces lanuginosus* increased the proportion of orthophosphates in hemp pomace. Orthophosphates, primarily existing as H₂PO₄⁻ and HPO₄^2^⁻ in soils, are highly reactive with elements such as calcium (Ca), magnesium (Mg), iron (Fe), and aluminium (Al). These interactions can lead to the precipitation of orthophosphates, rendering them insoluble. However, the observed increase in orthophosphate content suggests that *T. lanuginosus* may have enhanced the solubility of phosphorus compounds, potentially by increasing the solubility of orthophosphates while decreasing those of monoesters. This alteration could be attributed to the production of enzymes like phytase, known to hydrolyze phytate and release orthophosphate, thereby improving phosphorus availability^32^.

#### Influence of solid-state fermentation on the structure of hemp pomace lignin

Lignin was isolated from hemp pomace prior to and during SSF (after 4, 7 and 10 days of fermentation) and analysed by ^13^C CP MAS NMR spectroscopy. The samples are labelled as HopLIG, HopLIG 4, HopLIG 7 and HopLIG 10.

**Fig. 3:**
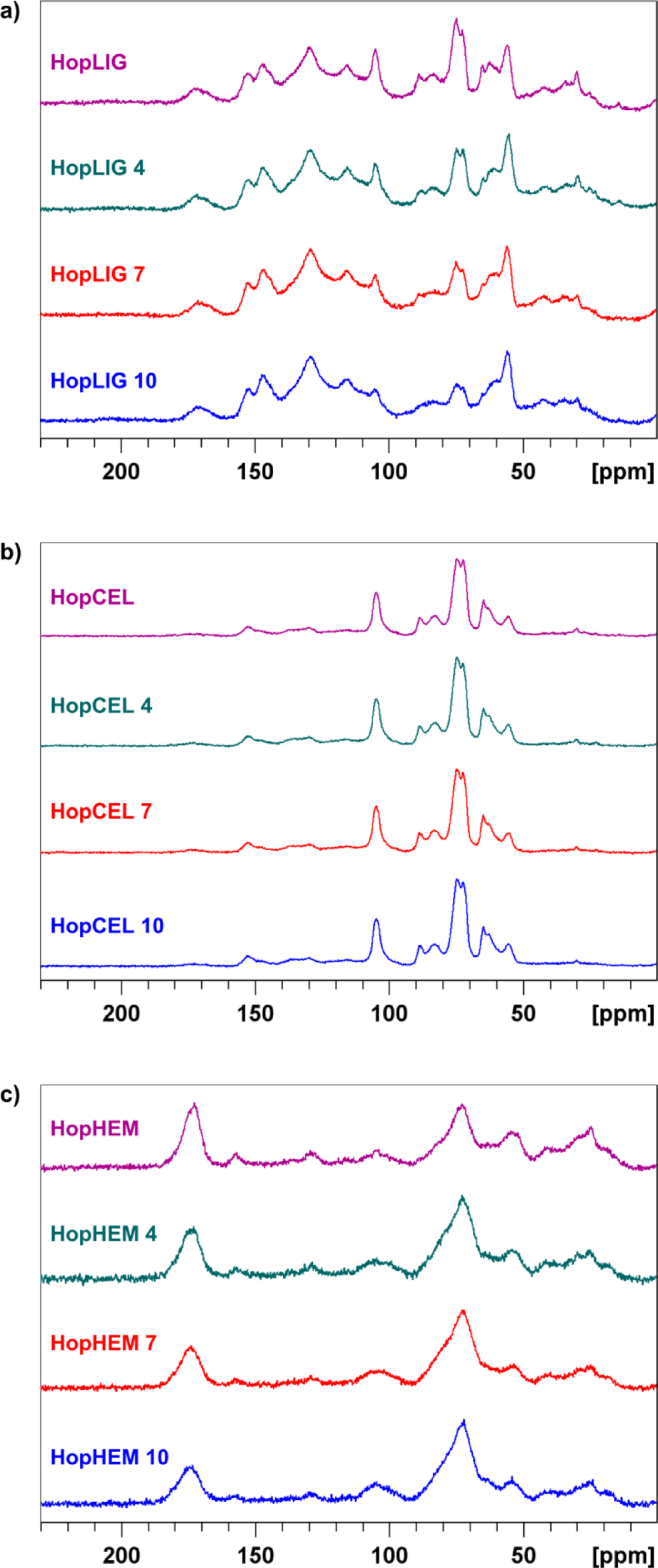
^13^C CP MAS NMR spectra of (**a**) hemp oil pomace lignin (HopLIG, HopLIG 4, HopLIG 7, HopLIG 10), (**b**) hemp oil pomace cellulose (HopCEL, HopCEL 4*,* HopCEL 7*,* HopCEL 10) and (**c**) hemp oil pomace hemicellulose (HopHEM, HopHEM 4*,* HopHEM 7*,* HopHEM 10).

The chemical structure of the lignin is presented in Table 3, while ^13^C CP MAS NMR spectra are presented in Fig. 3a.

**Table 3:** ^13^C CP MAS NMR assignments and percentages of the main functional groups in the lignin.

Composition / %	Hemp oil pomace lignin			
	HopLIG	HopLIG 4	HopLIG 7	HopLIG 10
Alkyl–C (0–45 ppm) / %	4.22	10.75	12.22	11.85
NCH/OCH_3_ (45–65 ppm) / %	16.26	17.70	17.67	18.66
*O*–Alkyl C (65–90 ppm) / %	25.15	16.68	13.15	10.92
O–C–O (90–110 ppm) / %	8.07	6.21	5.52	5.50
Aromatic–C (110–145 ppm) / %	32.14	31.79	32.76	36.22
Aromatic C–O (145–165 ppm) / %	13.07	13.89	14.82	14.74
COO/N–C=O (165–190 ppm) / %	1.09	2.98	3.86	2.11
Total Al–C (0–110 ppm) / %	53.70	51.34	48.56	46.93
Total Ar–C (110–165 ppm) / %	45.21	45.68	47.58	50.96

Lignin represents a valuable yet underexploited resource with significant potential for industrial applications. To fully harness lignin’s capabilities in the production of biofuels, bioplastics, adhesives, and other high value-added products, a comprehensive qualitative and quantitative characterization is essential. Such detailed analysis provides crucial insights into lignin’s complex chemical structure and variability, enabling the development of tailored processing methods and improving conversion efficiency in various biotechnological and chemical processes. It is known that lignin has the potential for the production of adsorbents, dispersants, flocculants for wastewater treatment, supercapacitor electrodes, bioplastics (hydrogels, thermosets, thermoplastics, foams and rubbers), lignin-derived chemicals (*e*.*g*. vanillin), films in agriculture, bio-additives, asphalt binders and controlled-release fertilisers^33^.

Although ^13^C CP MAS spectra are not fully quantitative, they can still provide valuable information on changes in the chemical composition of lignin. The NMR results of lignin prior and after the fungal treatment are presented Table 3. The lignin from hemp pomace contains a slightly higher proportion of aliphatic compounds and a lower proportion of aromatic compounds. However, these ratios have changed upon exposure to *T. lanuginosus*. Fungal treatment caused transformation of the material into a renewable source of aromatic feedstock, which could potentially be utilized in the chemical industry for the production of benzene, toluene, and xylene (BTX). Fermentation had the most significant effect on the alkyl–C and O–alkyl–C groups in lignin, increasing the content of alkyl–C while decreasing the content of O–alkyl–C groups. The hyperbranched polymer structure of lignin positions makes it as a potential dispersant. Our results have shown that fungal treatment increased the content of carboxyl groups, thereby enhancing the interaction with aqueous solutions. This finding aligns with previous studies where lignin was functionalized with carboxylic acid (− COOH) and amine (− NH_2_) groups, which improved its solubility^34^. Moreover, these results suggest that fungi could enhance the potential for producing polycarboxylic acids from hemp pomace. It is known that lignin can also be used in the pavement industry due to its structural similarity to bitumen^35^. The increase in content of carbonyl groups can improve the anti-aging properties of bitumen and this can be an additional benefit of the fungi. Generally, the effect of *T. lanuginosus* on lignin shows a tendency to increase the percentage of carbonyl groups.

#### Influence of solid-state fermentation on the structure of hemp pomace cellulose

The chemical composition of the cellulose samples is presented in Table 4, while ^13^C CP MAS NMR spectra are displayed in Fig. 3b.

**Table 4:** ^13^C CP MAS NMR assignments and percentages of functional groups in the cellulose.

Composition / %	Hemp oil pomace cellulose			
	HopCEL	HopCEL 4	HopCEL 7	HopCEL 10
Alkyl–C (0–45 ppm) / %	1.63	1.17	0.97	0.56
NCH/OCH_3_ (45–65 ppm) / %	20.36	22.47	22.70	22.96
*O*–Alkyl C (65–90 ppm) / %	55.79	55.35	55.15	55.46
O–C–O (90–110 ppm) / %	12.95	13.81	12.73	12.85
Aromatic–C (110–145 ppm) / %	6.13	4.66	4.95	5.17
Aromatic C–O (145–165 ppm) / %	2.92	2.19	3.01	2.80
COO/N–C=O (165–190 ppm) / %	0.22	0.35	0.49	0.2
Total Al–C (0–110 ppm) / %	90.73	92.77	91.55	91.83
Total Ar–C (110–165 ppm) / %*	9.05	6.88	7.96	7.97

Cellulose is a linear polysaccharide consisting of thousands of D-glucose units linked by β-1,4-glycosidic bonds. The analysis of the ^13^C CP MAS NMR spectra of cellulose isolated from hemp pomace revealed the presence of residual lignin. As shown in Table 4, the effect of *T. lanuginosus* on cellulose appears comparable to its effect on lignin. Specifically, an increase in the intensity of signals within the 45–65 ppm region was observed, accompanied by a reduction in signal intensity in the 60–90 ppm region. These changes suggest structural modifications in the polysaccharide matrix. The HopCEL sample initially contained over 90% aliphatic carbon, the proportion of which further increased following fungal treatment, while the aromatic carbon content decreased, meaning that the residual lignin or other aromatic compounds were further broken down or removed and the cellulose became more chemically homogeneous (less contaminated).

Cellulose serves as a fundamental raw material for numerous essential industrial products, including paper, cardboard, paperboard, absorbent cotton, and textile-grade cellulose fibres. In addition, cellulose derivatives—such as esters and ethers—are widely utilized in the production of paints, explosives, adhesives, films, and celluloid materials.

#### Influence of solid-state fermentation on the structure of hemp pomace hemicellulose

Chemical composition of hemicellulose samples is displayed in the Table 5. ^13^C CP MAS NMR spectra of hemicellulose samples are given in the Fig. 3c.

**Table 5:** ^13^C CP MAS NMR assignments and percentages of functional groups in the hemicellulose.

Composition / %	Hemp oil pomace hemicellulose			
	HopHEM	HopHEM 4	HopHEM 7	HopHEM 10
Alkyl–C (0–45 ppm) / %	18.58	17.36	15.69	3.44
NCH/OCH_3_ (45–65 ppm) / %	16.40	16.57	16.14	0.49
*O*–Alkyl C (65–90 ppm) / %	24.26	39.10	42.02	62.05
O–C–O (90–110 ppm) / %	0.77	7.00	7.51	4.89
Aromatic–C (110–145 ppm) / %	2.10	2.00	2.22	0.09
Aromatic C–O (145–165 ppm) / %	1.67	1.01	1.00	1.45
COO/N–C=O (165–190 ppm) / %	36.22	16.96	15.42	27.59
Total Al–C (0–110 ppm) / %	59.24	80.03	81.36	70.87
Total Ar–C (110–165 ppm) / %	3.77	3.01	3.22	1.54

Hemicellulose is a polymer usually made up of pentose and/or hexose sugars such as xylose, glucose, mannose, and galactose in the backbone as well as arabinose, galactose, glucose, and glucuronic acid in the branches^36^. The C5 and C6 sugars are linked together by 1,3-, 1,6- and 1,4-glycosidic bonds and by acetyl groups, creating a loose and hydrophilic structure that acts like an adhesive between cellulose and lignin. Hemicellulose can be used in various industries, such as medicine, pharmaceuticals, fuel, packaging and textile^37^.

NMR analysis of hemicellulose isolated from hemp pomace during SSF revealed a pronounced effect of fungal activity on its chemical composition. As shown in Table 5, the relative intensity of signals corresponding to methoxyl groups (≈ 60 ppm) decreased during the fungal treatment, suggesting partial demethoxylation likely associated with lignin-carbohydrate complex degradation. Concurrently, an increase in signal intensity within the 65–110 ppm region was observed, which is characteristic of carbohydrates such as xylans and mannans. This spectral shift indicates a relative enrichment of polysaccharide components within the hemicellulose fraction.

## Conclusion

The application of solution-state and solid-state NMR spectroscopy proved to be a powerful tool for monitoring the structural and compositional changes in biopolymers and lipids isolated from hemp pomace before and after the solid-state fermentation by *Thermomyces lanuginosus*. The fungal treatment led to substantial modifications in the chemical profile of the biomass, enhancing the functional quality of its individual components. Lipid analysis revealed a clear decrease in mono-, di-, and triglycerides over the 10-day fermentation period, accompanied by an increase in free glycerol and unsaturated fatty acids. The rise in glycerol content is particularly noteworthy, given its widespread industrial relevance across food, pharmaceutical, cosmetic, and bio-based chemical sectors. This suggests that fungal bioconversion of agricultural residues may represent a viable strategy for sustainable glycerol production. In lignin, an increase in carboxyl group content was observed, which not only suggests improved reactivity but also points to the potential for producing polycarboxylic acids—compounds of interest for biopolymer synthesis and functional material development. In the hemicellulose and cellulose fractions, an enrichment of carbohydrate-related signals (65–110 ppm) indicates a shift toward greater polysaccharide purity and reduced aromatic or methoxylated impurities, likely due to fungal degradation of associated lignin structures. Additionally, changes in the spectral features of phosphorus-containing compounds point to fungal influence on their solubility and speciation, which could have implications for nutrient recovery or biofertilizer development.

Overall, the research highlights the potential of *T. lanuginosus* for the valorization of lignocellulosic biomass such as hemp pomace. This study contributes to the expanding field of microbial biomass processing and supports the integration of fungal biotechnology into circular bioeconomy frameworks for the sustainable production of value-added compounds from lignocellulosic biomass.

## Supplementary Information

Below is the link to the electronic supplementary material.

Supplementary Material 1

## Data Availability

The data presented in this study are available on request from the corresponding authors.
