# Supplementary material for: NMR characterisation of biopolymers and lipids from hemp pomace treated with Thermomyces lanuginosus
Source: Sci Rep. 2026 Mar 13;16:13345. doi: 10.1038/s41598-026-41682-1 (PMC13106638; doi:10.1038/s41598-026-41682-1)
Supplement: Supplementary file 1 — Supplementary Material 1 [file 41598_2026_41682_MOESM1_ESM.docx]

Table S1. ^1^H NMR assignments of hemp oil pomace lipid extracts

| **Signal** | **Chemical shift /ppm** | **Protons** | **Chemical groups (compounds)** |
| --- | --- | --- | --- |
| **1** | 0.89 | –CH_2_CH_2_CH_2_–C**H_3_** | terminal –CH_3_ except linolenic acid |
| **2** | 0.9 | –CH=CH–CH_2_–C**H_3_** | terminal –CH_3_ of linolenic acid |
| **3** | 1.12–1.46 | –(C**H_2_**)_n_– | acyl groups and fatty acids |
| **4** | 1.53–1.74 | C**H_2_**–CH_2_–OCO– | acyl groups, triglicerides and fatty acids |
| **5** | 1.94–2.15 | –C**H**_2_–CH=CH | all unsaturated acyl chains and fatty acids |
| **6** | 2.24–2.40 | –C**H2**−OCO– | acyl groups, triglicerides and fatty acids |
| **7** | 2.76 | –CH=CH–C**H_2_**–  CH=CH– | di-unsaturated acyl groups and fatty acids |
| **8** | 2.79–2.84 | –CH=CH–C**H_2_**–  CH=CH– | polyunsaturated acyl groups and fatty acids |
| **9** | 4.07–4.41 | ROC**H_2_**–CH(OR´)– C**H_2_**OR´´ | glycerol group in triglyceride |
| **10** | 5.23–5.31 | ROCH**_2_**–C**H**(OR´)– CH**_2_**OR´´ | glycerol group in triglyceride |
| **11** | 5.31–5.53 | –C**H**=C**H**– | acyl groups and fatty acids |
| **a** | 3.65 | ROCH_2_–CHOH–C**H**_2_OH | glycerol groups in 1-MG |
| **b** | 3.73 | ROCH_2_–CH(OR´)–C**H**_2_OH | glycerol groups in 1,2-DG |
| **c** | 3.94 | ROCH_2_–C**H**OH–CH_2_OH | glycerol groups in 1-MG |
| **d** | 4.06–4.20 | ROC**H**_2_–C**H**OH–C**H**_2_OH | glycerol groups in 1,3-DG |
| **e** | 4.19 | ROCH_2_–CHOH–C**H**_2_OH | glycerol groups in 1-MG |
| **f** | 5.09 | ROCH_2_–C**H**(OR´)–CH_2_OH | glycerol groups in 1,2-DG |
